# Ancient DNA from lake sediments: Bridging the gap between paleoecology and genetics

**DOI:** 10.1186/1471-2148-11-30

**Published:** 2011-01-27

**Authors:** Lynn L Anderson-Carpenter, Jason S McLachlan, Stephen T Jackson, Melanie Kuch, Candice Y Lumibao, Hendrik N Poinar

**Affiliations:** 1 Department of Biological Sciences, University of Notre Dame, Notre Dame IN, 46556, USA; 2 Department of Botany and Program in Ecology, University of Wyoming, Laramie, WY, 82071, USA; 3 McMaster Ancient DNA Centre, Department of Anthropology and Biology, McMaster University, Hamilton, Ontario L8S 4L9, Canada

## Abstract

**Background:**

Quaternary plant ecology in much of the world has historically relied on morphological identification of macro- and microfossils from sediments of small freshwater lakes. Here, we report new protocols that reliably yield DNA sequence data from Holocene plant macrofossils and bulk lake sediment used to infer ecological change. This will allow changes in census populations, estimated from fossils and associated sediment, to be directly associated with population genetic changes.

**Results:**

We successfully sequenced DNA from 64 samples (out of 126) comprised of bulk sediment and seeds, leaf fragments, budscales, and samaras extracted from Holocene lake sediments in the western Great Lakes region of North America. Overall, DNA yields were low. However, we were able to reliably amplify samples with as few as 10 copies of a short cpDNA fragment with little detectable PCR inhibition. Our success rate was highest for sediments < 2000 years old, but we were able to successfully amplify DNA from samples up to 4600 years old. DNA sequences matched the taxonomic identity of the macrofossil from which they were extracted 79% of the time. Exceptions suggest that DNA molecules from surrounding nearby sediments may permeate or adhere to macrofossils in sediments.

**Conclusions:**

An ability to extract ancient DNA from Holocene sediments potentially allows exciting new insights into the genetic consequences of long-term environmental change. The low DNA copy numbers we found in fossil material and the discovery of multiple sequence variants from single macrofossil extractions highlight the need for careful experimental and laboratory protocols. Further application of these protocols should lead to better understanding of the ecological and evolutionary consequences of environmental change.

## Background

Knowledge of the long term population history of a plant species provides clues to its current distribution [1-5], abundance [2,6] and genetic structure, [7-11], and can also help assess its potential response to future environmental change [12,13]. Ecological and biogeographic history have been inferred primarily from paleoecological studies (fossil pollen and macrofossils in sediments of lakes and peatlands, middens accumulated by packrats and other rodents, tree-rings from living and dead trees) and phylogeographic studies (spatial patterns of molecular and other genetic markers in living populations). These records indicate that terrestrial plant species have undergone population expansion, contraction, isolation, fragmentation, and coalescence of previously isolated populations since the last glacial maximum [2-4,14,15].

Paleoecology and phylogeography each have unique advantages and liabilities in historical inference. Pollen assemblages incorporate pollen grains from a variety of local and distant sources [16], and morphological similarities frequently prevent taxonomic resolution below the genus or even family level [14]. Plant macrofossils offer greater taxonomic and spatial precision [17], but sample sizes may be small. Phylogeographic and population genetic analyses often rely on broad assumptions to infer population histories, but lack sufficient information to connect genetic indices like effective population size (*Ne*) to the census population size (*N*). Uniting the disparate fields of genetics and paleoecology could compensate for their respective weaknesses [18,19].

Most studies linking plant paleoecological data to measures of genetic change have relied on indirect links between independent datasets [10,11,15,20-22]. Quite a few studies take advantage of time series of genetic data provided by ancient DNA (aDNA), usually without explicitly linking them to changes in fossil data [23]. Studies directly linking ancient DNA (aDNA) to fossil material [24-28] can explicitly characterize the genetic consequences of observed fluctuations in census populations. Until now, these examples apply exclusively to changes in animal populations. Progress for plant species has been limited by technical difficulties in extracting aDNA from the sort of fossils commonly used in paleoecological studies, though recent advances in this area show promise [29,30]. Here, we introduce a new approach to aDNA extraction and sequencing: the genetic analyses of lake sediments and associated macrofossils.

The successful amplification of aDNA from a variety of materials suggests that identifiable DNA may occur in plant macrofossils and sediments from lakes. However, a wealth of experience from other studies, as well as trial and error from our own work, indicate that the search for aDNA must be approached with caution [31-33]. In waterlogged environments, such as those most common in Holocene fossil studies, hydrolysis of DNA molecules presents a special challenge [30], but there are notable successes in extracting plant DNA from waterlogged material [34-36]. Generally, DNA degradation, low DNA yields, and the risk of sample contamination by modern DNA have led researchers to develop strict protocols for aDNA extraction and amplification [32,33,37-39].

The purpose of our paper is to develop and evaluate a reliable and repeatable protocol for aDNA extraction, amplification, and sequencing from plant macrofossils and bulk sediment from small lakes typically used in paleoecological studies. We sampled macrofossils of four tree species from sediments spanning the last 8000 years from seven lakes with contrasting bathymetry and water chemistry in the western Great Lakes region. We also analyzed eight bulk-sediment samples from four of these lakes. We address the following questions: Can aDNA be reliably extracted from macrofossils and bulk sediments? Does preservation differ between sites, species, or ages? How much DNA can be extracted from fossil samples relative to the DNA yield in senescent plant tissue that initially falls into lakes? Do macrofossils contain exogenous DNA? And, to what extent are detailed aDNA protocols to prevent sample contamination necessary?

## Methods

### Field and Laboratory Sampling

**Figure 1:**
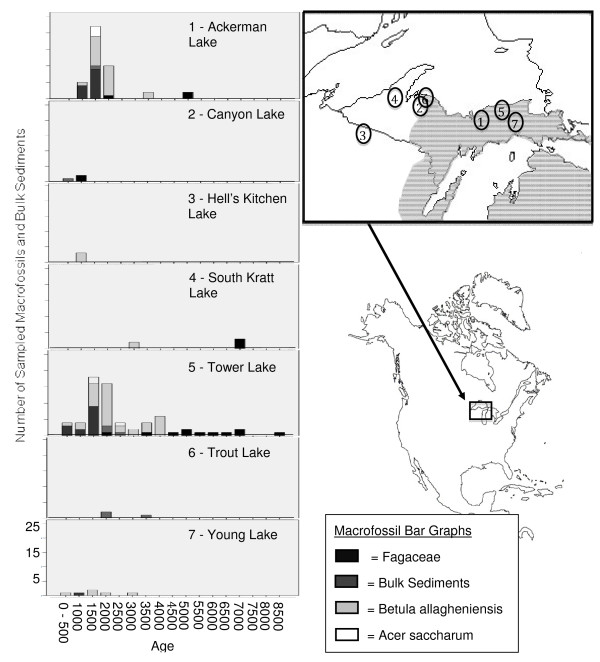
**Sample origins**. North American map indicating the location of sampled lakes (numbers). The modern range of beech is shaded in gray. The contemporary ranges of all other sampled species are throughout the sampled range. Bar graphs depict the number of macrofossils and bulk sediments taken from each lake's sediments through time.

Our samples come from unconsolidated sediments of seven small lakes in Upper Michigan and adjacent Wisconsin (Figure 1, Table 1). The upper 60-100 cm of sediment from Canyon, Trout, and Ackerman Lakes were obtained using a wedge- type freeze-corer [40,41]. Upper sediments (topmost 60-80 cm) from the other sites, as well as deeper sediments from Trout and Ackerman Lakes, were obtained using a 7.5-cm diameter modified Livingstone piston corer [42]. Cores were extruded in the field, wrapped in plastic film, and transported to the paleoecological laboratory at University of Wyoming where they were stored at 4°C. Since the cores were collected independently of our studies of aDNA, they were not coated with an exogenous DNA tracer (as per reference [43]). This raises the possibility of contamination by exogenous plant DNA during core processing. We partially compensated for this risk by sampling tissues and bulk sediment from interior portions of the core rather than from the core edge, where exogenous DNA could most easily contaminate the sediments. Cores were sampled in increments 1 cm in thickness, representing time-intervals ranging from 5 to 20 years per sample. All of the cores were rich in macrofossils, owing to small lake size and proximity of coring sites to steep upland slopes [17]. Sampling of materials for aDNA analysis was biased toward the last 3000 years, primarily because Jackson's ongoing paleoecological studies in the region were concentrated on late Holocene dynamics, so more material was available for study. Most of our samples (106/126) came from Ackerman or Tower Lakes (Figure 1, Additional File 1), which contained some of the richest and most diverse macrofossil records.

**Table 1:** Sample lakes (corresponding to Figures 1 and 6).

Lake Name	Lat/Long Coordinates	Surface Area (ha)	Maximum Water Depth (m)	Water Depth at Coring Site (m)
Ackerman Lake	46.33N/-86.790W	5	14	8

Canyon Lake	46.83N/-87.790W	1	23.7	23.7

Hells Kitchen Lake	46.19N/-89.7W	3	19	10

South Kratt Lake	46.84N/-88.860W	2.5	9	6.9

Tower Lake	46.54N/-86.040W	3	11	8.9

Trout Lake	46.86N/-87.890W	10	18	15

Young Lake	46.44N/-85.710W	2.5	9.5	8.7

All macrofossil and bulk sediment samples were extracted in the paleoecology laboratory at the University of Wyoming. Bulk sediment samples (1 cm^3^) were taken from the center of 1-cm slices of the sediment cores. Sediment samples for macrofossil analysis (10-100 cm^3^) were prepared by dispersal in distilled water, followed by sieving (710 μm mesh) under a stream of tap water. Sieve residues were rinsed and stored in distilled water and scanned under a stereomicroscope for macrofossil sorting. Macrofossils and bulk-sediment samples were placed in Falcon tubes after separation, and immediately frozen (-20°C) or freeze-dried. All instruments (forceps, sieves, vials, etc.) were sterilized between samples by flaming or bleach treatment. All samples were shipped to the aDNA laboratory at McMaster University in either a frozen or freeze-dried state.

Macrofossil samples included red oak (*Quercus rubra*), American beech (*Fagus grandifolia*), sugar maple (*Acer saccharum*), and yellow birch (*Betula allagheniensis*). Each species was typically represented by distinct tissue types. For example, samaras and catkin-bracts were most common for yellow birch, while beech was most commonly represented by bud scales (see Additional File 1). Macrofossil composition differed significantly over time and space (Fisher's exact test, p < 0.0001). These differences partially result from changing species' distributions over time (e.g., yellow birch and beech immigrated relatively late to the study area, and beech does not occur near our northeastern study sites).

Plant tissues reaching sediments range from living tissue to tissue that has persisted in aerobic conditions for long periods before being washed into the lake. To assess the yield (concentration) of aDNA extracted and amplified from our samples relative to this spectrum of tissues, we quantitated cpDNA in the type of senescent material that might wash or fall into a lake, as well as from analogous fresh tissues, using one 82-bp qPCR-assay. This allowed us to determine whether most DNA degradation/loss takes place after tissue senescence or after deposition in the sediments. Our senescent material consists of tissues from 10 individuals from the macrofossil reference collection at the University of Wyoming, which were all collected from live trees in the field, usually at the time of tissue senescence and/or shedding of organs. These samples were of the same organs as the plant macrofossils, and included seeds, bracts, and/or bud scales from American beech, sugar maple, and yellow birch. We did not have suitable reference samples of red oak, so we substituted bud scales from black oak, Gambel oak, and white oak (*Q. velutina, Q. gambelii, Q. alba*). Senescent tissues were original collected up to several decades ago, so DNA yield in these tissues reflects the original DNA content plus degradation during storage. Analogous fresh material was collected from trees on the University of Notre Dame campus. In the absence of a separate study of the DNA content of plant tissues arriving in sediments, we assume these end members bracket the likely DNA content of the source material reaching sediments.

### DNA extraction

DNA extraction protocols were the same for macrofossils, modern samples, and bulk sediments. All aDNA analyses were conducted at McMaster University's ancient DNA facilities, while modern analyses were conducted at facilities at the University of Notre Dame. Published aDNA protocols were used at McMaster including the division of sample preparation, extraction and PCR setup rooms; the use of protective clothing; and techniques to minimize the contamination risk including UV light exposure, bleach sterilization, use of PCR workstations, and filtered pipette tips [32].

Within the designated preparation room, samples were stored at -20°C until use. Immediately after removal from -20°C, samples were submerged into 600 μl CTAB buffer (2% CTAB; 2% PVP; 1.4 M NaCl; 10 mM EDTA, pH 8.0; and 100 mM Tris-HCl, pH 8.0) and chopped into very small pieces using sterile scalpels, after which they were taken to the extraction room for a 2 hour incubation at 65°C. Subsequently 600 μl of Phenol/Chloroform/Isoamyl alcohol (25:24:1) were added to each tube and tubes were vortexed and centrifuged at maximum speed (14,000 × g) for 5 minutes. The aqueous solution was transferred to another tube and 500 μl of CHCL_3 _was added. Samples were once again vortexed and centrifuged at maximum speed for 5 minutes. The resulting aqueous phase was removed for concentration using microcon cartridges (50 bp cutoff YM-10) (Millipore). Microcon membranes were first primed with 100 μl of 0.1× TE (10 mM Tris-HCl, and 1 mM EDTA, pH 8.0), after which samples were added and centrifuged at maximum speed (14,000 × g) for 10 minutes. Microcon membranes were then washed by adding 300 μl of 0.1× TE, pH 8.0, shaking at room temperature on an Eppendorf thermomixer at 650 rpm for 3 minutes and centrifuging at maximum speed (14,000 × g) for 10 minutes. To elute the DNA we added 100 μl of 0.1× TE pH 8.0 to the cartridges, which we then incubated at room temperature on a thermomixer at 1000 rpm for 5 minutes. Cartridges were then inverted into a new tube and centrifuged to acquire samples.

#### Negative and blank controls

We processed one negative extraction control for each six fossil samples extracted (total of 28 blank extraction controls) and followed these samples through PCR amplification. Negative extraction controls followed the identical procedure as aDNA extractions, minus the addition of fossil material. We ran 48 additional negative PCR controls, where PCR reagents were mixed without template DNA in the McMaster University cleanroom (prePCR) facilities, and then transported to a separate facility for PCR amplification. We highlighted the effectiveness of our strict protocols against contamination by supplementing these negative PCR controls with the type of negative controls typically used in molecular laboratories focusing on modern tissue. In these "weak" negative PCR controls, we added PCR reagents to sterile water in the same room in which PCR products are analyzed.

In addition to PCR and extraction controls, we processed nine extraction blank controls to test directly for modern DNA contamination along the DNA extraction pipeline. Eight water samples were obtained from sieving and sorting sediment residues from Ackerman Lake (4 samples) and Tower Lake (2 samples) and from tap water in the University of Wyoming's lab (2 samples). We also processed one ambient air sample (obtained from that lab) from a Falcon tube that was opened in his lab to test for airborne DNA particles. DNA extractions of these samples were conducted using the same extraction protocol as for aDNA samples. Positive aDNA controls in this analysis consisted of three macrofossils (two oak leaf fragments and a leaf fragment of yellow birch), which had amplified and quantified reliably in previous PCR reactions.

### DNA amplification

For all species, we amplified and sequenced a single 82-bp section of the spacer region between the coding genes atpB and rbcL in the chloroplast genome [44]. Sequence variation in this region within and between species has been characterized by extensive sampling throughout eastern North America (including the Upper Midwest) in our focal species as well as in other common eastern trees [15] and McLachlan unpublished. Polymorphisms in this fragment reliably distinguish three taxonomic groups among the species we investigated: Fagaceae (beech and red oak), sugar maple, and yellow birch, each distinguished by one 2 bp polymorphic site with three individual character-states for each group (See Table 2). We also detected within-group variation among Fagaceae through a transversion mutation. PCR amplification reactions consisted of 1× PCR buffer, 2.5 mM of MgCl_2_, 400 uM of dNTPs, 300 nM of each primer, 0.05 units of Taq Gold (Applied Biosystems), 1 mg/ml of BSA, 30 mM of Rox Dye as a reference dye, and 0.167 × of SybrGreen. Cycle conditions consisted of: 95°C-7 min initial denaturation, followed by 50 cycles of 95°C-30 sec, 56°C-30 sec, 72°C-30 sec, and a final extension of 72°C for 15 min. Primers used were Forward: 5'-ATTGGGTTGCGCCATACATA-3', Reverse: 5'-GGTTCGTTATTAGACCATGATATTTGAT-3'.

**Table 2:** Genetic variation within the 82 bp sequenced region in modern tissue of known species and unknown variants in fossil material.

Taxonomic ID [Genbank Number]	Mutations															
**Fagaceae [**HQ333118**] △**																
American Beech 1		G	G	C	A	A	T	A	A	G	G	T	T	G	C	G
Chestnut		G	G	C	A	A	T	A	A	G	G	T	T	G	C	G
Red oak		G	G	C	A	A	T	A	A	G	G	T	T	G	C	G
Northern Pin Oak		G	G	C	A	A	T	A	A	G	G	T	T	G	C	G
Pin Oak		G	G	C	A	A	T	A	A	G	G	T	T	G	C	G
Black Oak		G	G	C	A	A	T	A	A	G	G	T	T	G	C	G
**American Beech 2 [**HQ333119**] +**		G	G	C	A	A	T	A	**C**	G	G	T	T	G	C	G
**Sapindaceae [**HQ333120**] ×**																
Red Maple		G	G	C	A	A	T	A	A	**A**	G	T	T	G	**T**	G
Sugar Maple		G	G	C	A	A	T	A	A	**A**	G	T	T	G	**T**	G
**Betulaceae [**HQ333117**] ○**																
Yellow Birch		G	G	C	A	A	T	A	A	G	G	T	T	G	**A**	**A**
Paper Birch		G	G	C	A	A	T	A	A	G	G	T	T	G	**A**	**A**
Black Birch		G	G	C	A	A	T	A	A	G	G	T	T	G	**A**	**A**
**UNKNOWN 1 [**HQ333121**] ▽**	*	G	**A**	C	A	A	T	A	A	**C**	G	T	T	G	**T**	G
**UNKNOWN 2 [**HQ333122**] ◇**	*	G	G	**T**	A	A	T	A	A	G	G	T	T	G	**A**	**A**
**UNKNOWN 3 [**HQ333123**] □**		G	G	C	A	A	**A**	A	A	G	G	**A**	T	G	**A**	G
**UNKNOWN 4 [**HQ333124**] ✴**	*	G	G	C	A	A	T	A	A	**A**	G	T	T	G	**A**	**A**
**UNKNOWN 5 [**HQ333125**] **		G	G	:	:	:	:	:	:	G	G	T	T	G	C	G
**UNKNOWN 6 [**HQ333126**] **	*	G	G	C	A	A	T	A	A	G	G	T	T	**A**	**A**	**A**
**UNKNOWN 7 [**HQ333127**] ✡**	*	**A**	G	C	A	A	T	A	A	G	G	T	T	G	**A**	**A**
**UNKNOWN 8 [**HQ333128**] ⊞**	*	G	G	C	A	A	T	A	A	G	**A**	T	T	G	**A**	**A**
**UNKNOWN 9 [**HQ333129**] ⊗**		G	G	C	A	A	T	A	A	G	G	T	:	G	**A**	**A**
**UNKNOWN 10 [**HQ333130**] **	*	G	G	C	A	A	T	A	A	**A**	G	T	:	G	**A**	**A**
**UNKNOWN 11 [**HQ333131**] ■**	*	G	**A**	C	A	A	T	A	A	G	G	T	T	G	**A**	**A**
**UNKNOWN 12 [**HQ333132**] ◆**	*	G	G	C	A	A	T	A	A	**A**	G	T	T	G	C	G

Mutations are in bold. Symbols correspond with Figures 5 and 6. Asterisks indicate mutations that are consistent with potential DNA damage.

### Amplification success

Amplification success (indicated by the presence of a band on an agarose gel, or a successful qPCR reaction) could be influenced by several factors, including differential DNA survival over time or in different lakes, primer specificity, PCR inhibition, etc. We fit a binomial generalized linear model (GLM) to amplification success per sample with species identity, lake of origin, tissue type, sample age, and number of PCR attempts as explanatory variables. To more flexibly estimate the relationship of amplification success to sample age, we fit a binomial general additive linear model (GAM) to the success data with age as a covariate. All statistical modeling was conducted using R (http://www.r-project.org).

### DNA yield

We used qPCR reactions to test for primer sensitivity and efficiency of the reaction by amplifying a dilution series of known DNA copy numbers (31250, 6250, 1250, 250, 50 and 10 copies/5 μL). High R^2 ^values (> 0.994) in a regression of the number of DNA copies against PCR cycle at a standardized threshold (Ct) provide evidence that our reaction is efficient and, as the low end of the standard amplifies well, that the reaction is also sensitive. Quantitative PCR is thus used to ensure that our estimates of DNA quantity are reliable, even for smaller DNA quantities. We then quantified DNA yields (in copy numbers) of our ancient samples by comparing amplification plots of the samples to those of the standard dilution series [45]. We used Fisher's exact test to assess the efficiency of our reactions at low copy number: we predicted that samples that amplified with low DNA yield would also amplify in a second quantitation attempt and that samples that had previously failed to amplify would not amplify in this second attempt.

We tested whether DNA quantity (calculated as number of DNA copies 5 μl^-1^) was influenced by the same variables we analyzed for PCR success (sample age, species, lake of origin, and number of attempted PCR reactions) using a GLM with lake and species as categorical variables. We ran a separate GLM to find out whether bulk sediments or macrofossils yielded higher DNA concentrations with sample age, lake of origin and number of attempted PCR reactions as covariates. For all analyses of yield, DNA quantities were log transformed to normalize the data (*ln*DNA).

The PCR reaction can be inhibited by the coeluates of extraneous compounds (e.g., tannins) in the DNA extracts as well as damage to the DNA molecule in the form of covalently bound peptides or crosslinks [43]. Such inhibition can reduce estimates of total amplifiable DNA. We tested for the presence of inhibition by incorporating a mammoth (*Mammuthus*) cytB DNA standard into our qPCR analysis [24]. Mammoth DNA is unlikely to have been present in any of our relatively young lake sediment samples. By adding known quantities of a PCR product derived from mammoth mtDNA into qPCRs containing our macrofossil and bulk sediment extracts, any deviation from the expected mammoth values during qPCR amplification would indicate inhibition from our aDNA extracts. We amplified 250 copies of a mammoth amplicon in the presence of 10 representative extracts, as well as 1:10 dilutions of each extract. In the absence of polymerase inhibition, these reactions should amplify at the same Ct as the reaction with 250 copies of mammoth DNA without added extracts. Polymerase inhibition would be expected to increase the Ct of samples, and we expect that concentrated extracts would amplify at higher Ct than the diluted extract from the same sample in the presence of polymerase inhibition.

To illustrate the risk of contamination in more typical laboratory settings, we supplemented the four true negative controls in this PCR run with an additional four "weak negative controls" (see *negative and blank controls*). PCR amplification reactions consisted of 1× PCR buffer, 2.5 mM of MgCl2, 400 uM of dNTPs, 300 nM of each primer, 0.05 units of Taq Gold (Applied Biosystems), 0.75 mg ml^-1 ^of BSA, 30 mM of Rox Dye as a reference dye, and 0.167 × of SybrGreen. Cycle conditions consisted of: 95°C-7 min initial denaturation, followed by 45 cycles of 95°C-30 sec, 60°C-30 sec, 72°C-90 sec, and a final extension of 72°C for 15 min.

Finally, we wanted to identify the point(s) along the taphonomic process where DNA loss occurs. We did this by looking at DNA yields between fossil, modern senescent, and living material. Using the same protocols described above, we extracted DNA and quantified DNA yield from ten living and ten modern senescent tissues. We used a t-test to compare ancient *ln*DNA yields against modern senescent *ln*DNA yields.

### Phylogeny

Following the cpDNA phylogeography literature (e.g., Petit et al 2003), we refer to each sequence variant as a "haplotype". We constructed a cladogram of aDNA haplotypes in our samples using TCS (version 1.21)[46]. TCS calculates a pairwise distance matrix between sequence variants and calculates the probabilities for the mutational steps detected using frequency data and parsimony criteria (as defined in [47]). Using a 95% cutoff, a graphical output is generated representing the relationships between the haplotypes.

### Multiple haplotypes from single samples

Multiple sequence variants derived from a single PCR reaction could indicate DNA damage, or the different sequences could be derived from multiple species or mixtures of individuals of the same species with different haplotypes. DNA damage is difficult to ascertain with certainty. Here, we consider polymorphic sequences to be more likely the result of DNA damage if they differ from sequences identified in living samples by only one base-pair difference and if that difference is a G to A or a C to T transition [48]. Because we don't know which of these transitions represent DNA damage and which might be true polymorphic variation, we conducted analyses relevant to genetic composition and relevant to the predominance of multiple sequences twice: once including all sequence variation (assuming no DNA damage) and once excluding sequences that differ from known sequences by a single transition (assuming that all these sequences indicate DNA damage). Differences between the results of these paired analyses indicate the extent to which DNA damage might alter the interpretation of our results.

To detect multiple haplotypes within a single PCR product, we cloned the products using a Topo TA cloning kit (Invitrogen) as per manufacturer's instructions and sequenced 4 clones on average. To ensure that the consensus we derived for the remaining sequences were authentic we directly sequenced representative PCR products multiple (at least two) times from two different PCR reactions. We failed to produce a second PCR for eight of the 66 samples which had previously amplified. For these eight samples, we either only have one direct sequence or clones from a single PCR reaction (see Additional File 1). For cloning, bacterial colonies were grown on LB-agar plates and then lysed using a boil-prep protocol. Samples were then PCR amplified (1× PCR buffer, 2.5 mM MgCl_2_, 0.2 μM PrimerF, 0.2 μM PrimerR, 0.2 mM dNTPs, 1 U AmpliTaq Gold) under the cycling conditions of 94°C-5 min, (94°C-30 sec, 57°C-30 sec, 72°C-30 sec) 29 times, and a final 72°C extension for 15 minutes. In preparation for BigDye cycle sequencing, all colony-PCR products were purified using Millipore plates (YM-30), and all PCRs subjected to direct sequencing were cleaned using ExoSap (USB Scientific) according to manufacturer's protocols. BigDye cycle sequencing was conducted according to manufacturers protocols (Version 3.1), and reactions were run on an ABI 3730 for data collection.

We often found more than one cpDNA sequence among colony PCR products from the same reaction and also among separate direct PCR reactions from the same sample. We predicted that we would find multiple haplotypes more commonly in bulk sediments than in macrofossils. We conducted analyses first including all sequences (assuming no DNA damage), and then excluding sequences with polymorphisms associated with DNA damage. We used Fisher's exact test to determine if the probability of multiple haplotypes was greater in extractions from bulk sediments. We also tested this prediction with a logistic regression on the probability of multiple haplotypes in one sample extract with bulk sediment vs. macrofossil and the number of PCR attempts as explanatory variables. We also predicted that we would see *a higher number *of distinct haplotypes in bulk sediments than in macrofossils. We used a Poisson regression to test whether the number of haplotypes differed between DNA extractions from bulk sediments and macrofossils.

To the extent that we can distinguish taxonomic groups using developed molecular markers, we assessed whether the most common ('dominant') haplotype found in each individual macrofossil corresponded to that macrofossil's morphologically based taxonomic assignment. Our aim was to determine whether the dominant haplotype was more likely to represent endogenous DNA (i.e., from tissues of the macrofossil) rather than exogenous DNA (from sediment adhering to the macrofossil or from sample contamination). This analysis requires that we assign each sequence as correctly or incorrectly matching the macrofossil species identity. Unknown sequences, those that either did not correspond to any sequence in our modern data set or had ambiguous base-pair reads at sites that are polymorphic in modern samples, were rare (34/298 sequences). To conservatively bias our test, we coded these sequences as *incorrectly *matching macrofossil ID. This classification only altered the dominant haplotype code (to "incorrect") in one sample (of 57) and did not influence the significance of terms in our model. We modeled the probability that the dominant haplotype from each macrofossil corresponded to the taxonomic identification of that tissue as a binomial response variable. Predictor variables included in a generalized linear regression were number of sequences, number of attempted PCR reactions, DNA quantity, and age of samples. To assess the possible impact of DNA damage on our results, we conducted this test first using all sequences, and then repeated the test without the suspect sequences (WOSS).

### Changing genetic composition

As described above, species distributions and abundances have shifted over the last 5000 years at our study sites. For instance, beech macrofossils appear in our dataset only within the last 1500 years. We used pairwise Fisher's exact tests to test if the genetic composition of the fossil material we studied also changed over time. We divided our samples into three time periods with roughly equal sample sizes (0 - 1200 yr BP; 1200 - 1700 yr BP; and > 1700 yr BP) and compared relative haplotype frequencies between the oldest and youngest periods. Again, we conducted this test first under the assumption that no sequence variation was due to DNA damage, then excluding samples with polymorphisms suggestive of DNA damage (WOSS).

## Results

### Efficiency and reliability of our approach

Numerous checks and controls illustrate the effectiveness of our protocols for extracting and amplifying low-copy DNA while controlling for contamination by exogenous DNA. The tight match between predicted qPCR threshold cycle number and known DNA quantity in our dilution series (R^2 ^> 0.994) showed that the PCR reaction was sensitive and efficient and that even low numbers of DNA molecules could be amplified. In repeated runs of this dilution series throughout the experiment, however, the most dilute samples (10 copies (5 μl)^-1^) occasionally failed to amplify, presumably due to the stochasticity of PCR reactions with low template. Thus, we were not always able to reliably detect the lowest concentrations of DNA molecules.

**Figure 2:**
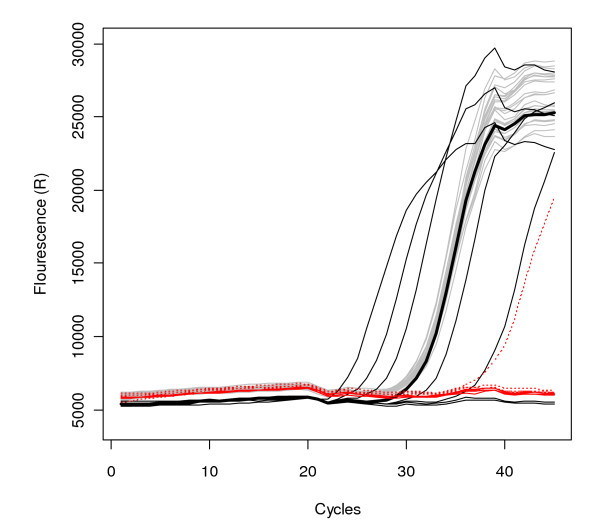
**Inhibition quantitation assay**. Graph depicts fluorescence values against cycle of appearance along a 45 cycle quantitative PCR reaction. Gray lines are 10 samples from our ancient DNA extracts and 10 1:10 dilutions of those samples, all spiked with a 250 copy standard. Black lines are the dilution series of mammoth DNA from 31,250 copies to 0.4 copies, with the 250 copy standard highlighted in bold. Solid red lines are negative controls and dashed red lines are our "weak" negative controls, used here to illustrate the risks associated with not separating pre- and post-PCR processing.

We found no evidence of PCR inhibition in our extracts. The amplification of known quantities of spiked mammoth DNA were not inhibited by our DNA extracts in twenty independent qPCR reactions (Figure 2). This result imparts confidence in our ability to detect and quantify the small amounts of DNA in our ancient samples.

An array of 85 negative controls indicated that low template samples are unlikely to result from contamination by ambient exogenous DNA. Quantitative PCR did not reveal DNA in the nine extraction blank controls of ambient water and air samples from the macrofossil extraction facilities at the University of Wyoming, despite a sensitivity of 10 to 50 DNA copies (5 μl)^-1^. Of 28 negative extraction controls and 48 negative PCR controls employed in the ancient DNA laboratory at McMaster University, we found one contaminated extraction negative control. The DNA sequence of this contaminant matched the species of the sample that was consistently placed next to it along the extraction process. (All samples are extracted in a particular order of alternating species to track potential contamination). It is likely that the single contamination event resulted from pipetting error, rather than contaminants in the laboratory environment. Of the four "weak" negative PCR controls we used to illustrate the risk associated with not separating low-template DNA preparation from PCR processing facilities, one was contaminated with approximately ten copies of DNA (Figure 2), illustrating the risks of weak controls when working with low-copy DNA template.

### PCR amplification success

**Figure 3:**
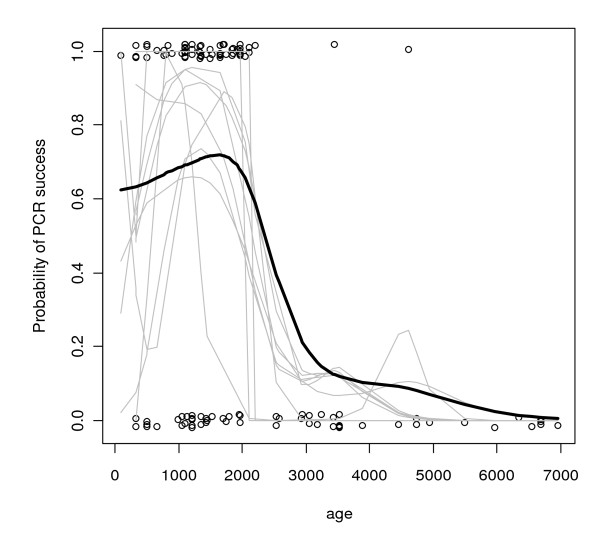
**Probability of PCR success**. Results of the binomial general additive model (black curve) comparing PCR success (1) to failure (0) relative to sample age. Data are slightly 'jittered' to reduce overlap of points. Gray curves are simulations in which data were thinned to an equal sampling effort.

PCR amplification success rate for samples less than 2000 years old was 64%, but the best fit of our binomial general additive model (Figure 3) indicates a sharp decline in successful amplification at this time. This dropoff in amplification success might be confounded by a coincidental decrease around this time in sampling intensity and in the number of PCR reactions we attempted for each extraction. This leaves open the possibility that older samples might have a relatively higher PCR success rate with more effort. However, we attempted to account for unequal sampling effort across time and unequal number of PCR attempts per sample by simulating samples of equal effort. In ten simulated datasets, we randomly thinned the data to a uniform sampling distribution across time and substituted a Bernoulli draw from the relative probability of success (number of successful PCR reactions/number of PCR attempts) for each individual's success. The thinned data are noisier, as we would expect, but confirm a drop in amplification success around 2000 yr BP independent of sampling effort (Figure 3).

We used a binomial generalized linear regression to confirm that the probability of successful DNA amplification decreased significantly (p < 0.001) with age. However, the confounding decline in the number of attempted PCR reactions was also significant (p < 0.001), which means that we are unable to discriminate the effect of age on PCR success from that of sampling effort in this analysis. We therefore accounted for unequal sampling effort across time by simulating samples of equal effort. In ten simulations, where data were thinned to a constant sampling effort across time and number of PCR attempts per sample, age significantly affected amplification success (p < 0.001), confirming a decline in PCR success in older samples. The binomial GLM also tested whether PCR amplification success could be explained by lake of origin, species, or whether DNA extractions were from macrofossils or bulk sediments. We found that amplification success was independent of lake of origin. Within the limits imposed by our sampling strategy, it appears that tissue type, species, and sedimentary environment do not strongly influence our ability to detect DNA in samples less than 2000 years old.

### DNA yield

**Figure 4:**
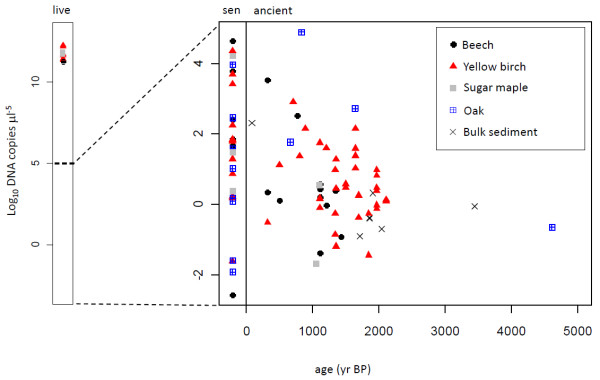
**DNA yield**. DNA quantity extracted from modern live material (live), senescent material (sen), and macrofossils and bulk sediments (ancient).

Among the samples with successful DNA amplification, DNA yield for the 82-bp cpDNA region was generally low for both macrofossils and bulk sediments (Figure 4). Our median DNA yield across all samples was < 10 copies (5 μl)^-1^, though aDNA yields also reached as high as 76,060 copies of DNA (5 μl)^-1^. We note that DNA quantitation was a good predictor of sequencing reaction success, even for samples with low DNA yield.

Of samples that quantitated with <10 copies (5 μl)^-1 ^in a final quantitation assay of all our DNA extracts, 39/41 had previously produced unambiguous sequence data, while samples that did not amplify in this reaction (68 samples) had usually failed to produce sequence data in previous positive PCRs (though 8 had previously produced successful sequences). A Fisher's exact test shows highly significant association (p < 0.0001) between the probability of a reaction failing or succeeding (despite low DNA yield) in the final pPCR reaction given results of previous reactions. This result supports our finding (reported in *Efficiency and reliability of our approach*, above) that even exceptionally low DNA concentrations are reliably amplified with the protocols presented here.

A GLM showed that the quantity of DNA extracted decreased significantly on a log scale with increasing age (p < 0.01). *ln*DNA yield also varied significantly among species (p < 0.01), with oak and yellow birch samples having significantly more amplifiable DNA than other sampled species. Increasing the number of PCR attempts also resulted in significantly higher DNA yield. In a second GLM, we found macrofossils had significantly more DNA than bulk sediments (p < 0.05).

DNA yields from ten samples of modern live material analogous in weight and tissue type to our ancient samples were more than six orders of magnitude larger than the highest yield from senescent or ancient material (Figure 4). Plant tissue arriving in sediment, however, originates from both living material and senescent material, including material that might persist in aerobic conditions for years before being washed into sediments. We found the average DNA yield from senescent modern material was significantly greater than average aDNA yield (t test: p = 0.04, 3.6031 ± 0.7747 SE, and 1.5115 ± 0.4195 SE, respectively), but the range of yields overlap broadly, with DNA yields from senescent tissue ranging from < 10 copies (5 μl)^-1 ^to ~ 43,000 copies (5 μl)^-1^, and aDNA yields ranging from < 10 copies (5 μl)^-1 ^to ~ 76,000 copies (5 μl)^-1 ^(Figure 4). Thus, while we can say that over 99.9999% of living tissue DNA is lost through taphonomic processes, we can not say what proportion of this loss occurs within the aquatic environment.

### DNA sequence data

**Figure 5:**
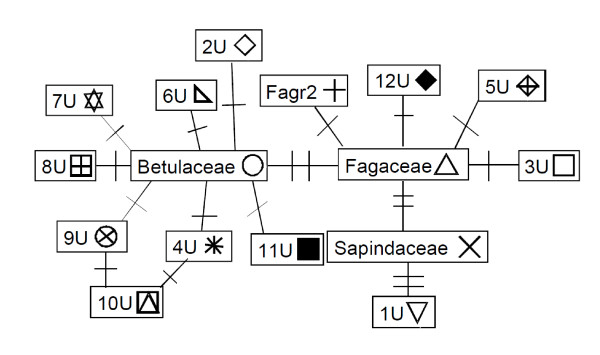
**Sequence variation**. Cladogram representing the relationship between haplotypes. Each Symbol represents a different haplotype. Each mutational step is represented by a hash mark. Cladogram symbols and identifications correlate with Table 2. Cladogram was constructed using TCS [46].

We successfully sequenced ancient cpDNA across multiple species, lakes, and ages, for both macrofossils and bulk sediment samples (see Additional File 1, or GenBank HQ333117 - HQ333132). Despite the small size of our cpDNA fragment, we identified 16 haplotypes in sediments, though only four of them matched sequences from known taxa (Table 2, Figure 5). Of all samples that amplified and were subsequently sequenced, 50% of the sequences were consistent with *Betula spp.*, 29% were consistent with Fagaceae, 0.003% were consistent with *Acer saccharum*, 10% were of unknown origin, and 10% had at least one ambiguous base. Seven of twelve unknown haplotypes were differentiated from the haplotype of a known species by a single transition (G to A or C to T), a pattern consistent with DNA damage (see Table 2). These sequences make up less than ten percent (30/310) of all unambiguous sequences. We cannot say with confidence whether these haplotypes represent DNA damage, error in the PCR reaction, or real but unidentified polymorphic variation in plants. We highlight the potential impact of DNA damage on relevant analyses below by repeating statistical tests without suspect sequences (WOSS).

Multiple haplotypes were obtained from single extractions from both macrofossils and bulk lake sediments. The frequency of multiple haplotypes in DNA extracts from macrofossils (0.45) was not significantly lower than the frequency of multiple haplotypes in bulk sediment samples (0.57) (Test of equal proportions, p = 0.86). When haplotypes suggestive of DNA damage are removed (WOSS), the frequency of multiple haplotypes decreases strongly in macrofossils (0.08), but it also decreases in bulk sediments (0.33) and these frequencies are not significantly different (p = 0.11). However, we attempted more PCR reactions from macrofossils than from bulk sediments. When we standardized for effort, the probability of sequencing multiple haplotypes from a single macrofossil was still not statistically distinguishable from the probability of sequencing multiple haplotypes from bulk sediments. When this analysis was repeated after removing haplotypes suggestive of DNA damage (WOSS), however, bulk sediments were shown to be more likely to produce multiple haplotypes than macrofossils (p < 0.05). This is the only case where excluding haplotypes consistent with patterns of DNA damage (WOSS) changed the significance of a statistical analysis. Neither the number of sequences recovered nor any of our usual explanatory variables (species, age, lake of origin, number of PCR attempts, bulk/macrofossil) significantly explained the number of haplotypes found in our samples in a Poisson regression (p > 0.05; WOSS, p > 0.05).

In macrofossil samples with at least two sequences, the dominant (most common) haplotype was consistent 80% of the time with the morphological identification of the source macrofossil (45 out of 57 individuals; WOSS, 45 of 53; see Additional File 1). This suggests that endogenous DNA in macrofossil tissue is amplified more often than ambient exogenous DNA either because it is present in higher quantities or because it is better preserved. None of the variables we used to predict the probability that the dominant haplotype correctly matched the macrofossil's taxonomic ID (tissue type, sample age, DNA yield, number of attempted PCR reactions, or the number of sequences examined) were significant in our binomial regression. The same non-significant results were found using WOSS data.

In summary, finding multiple haplotypes in DNA extractions from macrofossils and finding evidence of DNA damage both present technical challenges in our results, but most sequences in our dataset did not have these problems. Of the 57 macrofossils with unambiguous sequences, 40 (74%) had only one haplotype. Of these single haplotype samples, 35 (88%) had haplotypes matching the species ID of the macrofossil in question and the remainder had haplotypes matching other species. Of the 14 macrofossils with multiple haplotypes, 9 (64%) contained a mixture of the "correct" sequence and sequences that differed from this "correct" sequence by a single transition. Such samples appear to contain DNA from a single individual that has received a degree of DNA damage over the years. Of the remaining multi-sequence macrofossils, four had a mixture of "correct" sequences and sequences of other species and one had a mixture of sequences from other species. Additional File 1 classifies macrofossils according to these categories of damage and multiple haplotypes per sample.

### Changes in genetic composition over time

**Figure 6:**
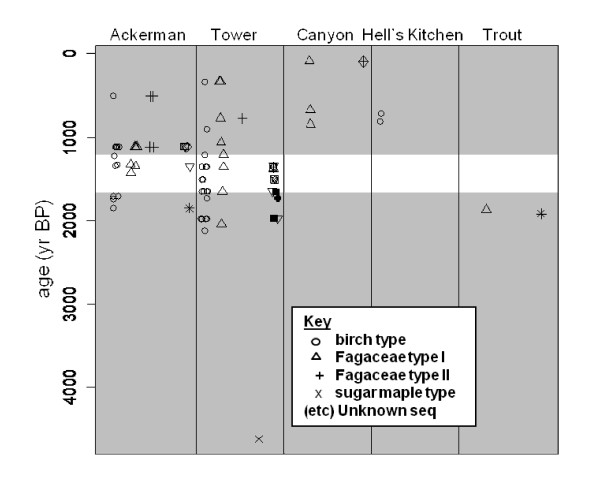
**Shifts in genetic composition**. Sediments less than 1200 years old (upper grey band) are genetically distinct from sediments over 1700 years old (lower grey band). This trend holds in comparisons limited to Ackerman and Tower Lakes, with samples spanning both time periods.

We found a significant difference between the genetic composition of young (<1200 yr BP) and old (>1700 yr BP) material (Fisher's exact test using all sequences, p = 0.01; WOSS, p = 0.02); (Figure 6). Younger sediments contained a higher proportion of Fagaceae haplotypes, consistent with the abundant beech budscales found only in younger sediments. This genetic change over time is potentially confounded by the inclusion of locations with only older sequences (Trout Lake) or only younger sequences (Canyon Lake, Hell's Kitchen Lake). We reran the analysis using only samples from the two sites that had relatively abundant sampling in both periods, Ackerman Lake and Tower Lake, which are located 100 km apart in the eastern Upper Peninsula of Michigan (Figure 1). There is no significant difference between the genetic composition of material in these two locations when samples are pooled across all time periods (p = 0.61; WOSS p = 0.45), but younger sediments pooled across the two sites contained material with a different genetic composition from older material (p = 0.02; WOSS p = 0.01). We found no significant differences between the genetic composition of old and young sediments of individual lakes or in pairwise comparisons between individual lakes, but sample sizes for these tests were small, so tests are unlikely to identify significant differences.

## Discussion and Conclusions

A recent review [49] argued that opportunities for aDNA recovery from sediments in temperate climates are likely to be worse than those in permafrost environments. Indeed, ancient plant DNA as old as 300,000 to 400,000 years old has been recovered from permafrost [43]. However, DNA preservation, degradation and leaching in permafrost settings are likely to be very different from that in lacustrine sedimentary environments and we want to be careful about apples to oranges comparisons. Successful amplification of plant DNA in temperate anaerobic sediments has, until now, been difficult. Holocene aDNA sequences have been recovered from macrofossils [50] and from individual pollen grains [51] in anaerobic peats and lake sediments, but success rates were very low (<10%) in these studies, even for recently deposited material. In our study, success rates are higher than previous analyses and inhibition is lower. On the other hand, the concentration of DNA extracted in our work is often extremely low, highlighting the importance of careful protocols against contamination. Comparisons in results between existing studies are ultimately somewhat weak, however, because each study sampled different tissues from different environments.

Application of aDNA data to the same plant tissues from the same depositional assemblages used in most temeprate paleoecological work has potential for greatly advancing and amplifying understanding of the ecological, evolutionary, and biogeographical consequences of the dramatic environmental changes of the recent past [18,19,29] further informing assessments of risks and appropriate actions in the face of rapid environmental change [11,52,53]. Our study constitutes a critical first step towards the goal of aDNA application to terrestrial plant paleoecology and biogeography. We document that small amounts of cytoplasmic DNA of multiple species can be extracted and amplified from materials, including a variety of plant macrofossils, preserved in Holocene lake sediments. The vast majority of DNA from living plant tissue appears to be lost before it reaches lake sediments and there is a further slower decline in DNA yield after sediment burial. Under rigorous protocols and safeguards, aDNA from lake sediments can be reliably extracted, amplified, and sequenced with a low threat of contamination by modern DNA. Despite the DNA depletion in sedimentary material, quantitative PCR allowed us to confidently produce sequence data after millennia of its burial in sediments.

Successful extraction and amplification from sedimentary materials younger than 2000 yr BP indicates that further applications to the late Holocene should be straightforward under our protocols. The steep decline in success beyond 2000 yr BP remains enigmatic, and deserves additional study. Age-dependent aDNA degradation processes may be responsible, but there is no obvious mechanism for rapid loss at the 2000-year threshold. A regional environmental signal is suggested by the simultaneous decline in PCR success across lakes. Paleohydrological and paleoecological studies in the region indicate that effective moisture increased substantially during the late Holocene (between 4000 and 2000 yr BP) [54-56]. Water-levels in all of our study lakes were probably substantially shallower before at least 3000 yr BP, and hence the sediment surface when early to mid-Holocene macrofossils were deposited was better-ventilated and perhaps more conducive to rapid DNA degradation. This hypothesis can be assessed with more-intensive sampling of older sediments, particularly comparing lakes and coring sites of differing water depths.

DNA extractions from most bulk-sediment samples and from some individual macrofossils contained mixed sequences representing multiple individuals and/or DNA damage. The occurrence of aDNA from multiple individuals is unsurprising for bulk sediments, which integrate diverse material from multiple sources (pollen, plant tissues, organic flocculents). Ancient DNA from multiple sources in individual plant macrofossils, particularly representing taxa other than that of the macrofossil itself, may derive from small sediment particles clinging to the macrofossil surface or trapped in small cracks. These sources are difficult to exclude in sample preparation; the rough microtopography of many macrofossil surfaces, as well as the frequent occurrence of tears or fissures, provide opportunity for materials from the surrounding sediment matrix to attach or infiltrate. Complete removal of these by either chemical or mechanical treatment before DNA extraction may be difficult without risking damage to the tissues and the aDNA contained within. We are now experimenting with alternative treatments to minimize extraneous aDNA from sediment matrix while also minimizing damage to the material and aDNA of interest.

Most phylogenetic and population genetic analyses, including those used in other aDNA studies [25], assume that the number of sampled individuals is known. Single macrofossils containing multiple haplotypes can hamper such applications. The aDNA sequences endogenous to individual macrofossils might be probabilistically distinguished from exogenous sequences; endogenous DNA dominated the mixture of sequences in most macrofossils. Additional evidence based on mechanism of DNA damage can be used to statistically identify sequence variation caused by DNA damage [57]. Distinguishing between the genetic identity of individual macrofossils, DNA damage, and the genetic composition of the sediment matrix, even in a probabilistic sense, would allow us to apply standard population genetic analyses to macrofossils (e.g., Beast, Serial SimCoal).

Applications of aDNA to macrofossils and other materials from lake sediments will require careful consideration of taphonomic issues, including the sources of the aDNA. Plant macrofossils in sediments of small lakes generally represent trees growing within 10^0^-10^1 ^meters of the lake margin [17,58]. Such spatial precision has considerable advantages in application, but it also ensures that only a small number of individual trees are likely to be represented in a macrofossil assemblage. DNA studies of surface sediments, linked with genetic census of trees near the lake margin, can help assess these and other issues of sampling and representation. DNA from pollen, whether analyzed from individual pollen grains [51] or integrated into bulk-sediment samples, represents a broader range of sources. Pollen assemblages in lake sediments comprise a distance-weighted integration of surrounding vegetation; although local individuals will be better represented than those farther away, a substantial amount of the pollen deposited in small lakes derives from plants growing 10^2^-10^5 ^m from the lake margin [16]. Bulk sediments may contain plant aDNA from a variety of sources, including pollen and plant tissues. Comparison of DNA signatures from bulk surface sediments with genetic data from multiple spatial scales may help identify the potential sources. Genetic signals at different spatial scales, for example, might be extracted using a combination of macrofossils and pollen grains as aDNA sources.

The degree of temporal integration or smoothing in aDNA signatures is also of concern. Our lake-sediment samples each represented narrow intervals of deposition (5-20 years), although deposition rates can be far slower in many lake settings [59]. Once entombed, individual plant macrofossils and smaller particles, including pollen grains, are unlikely to move vertically. Although vertical migration or leaching of DNA has been observed in sediments of caves, [60], we view this as highly unlikely in lake sediments. Periodic downward percolation of water can evidently move DNA in porous, granular sediments of caves and perhaps soil profiles. Lake sediments, in contrast, are permanently saturated and gravitational percolation of water does not occur. Once sediments are compacted, vertical advection of pore water is minimal, and multivalent metals and organic compounds (pigments, polycyclic aromatic hydrocarbons (PAHs), organic molecules with more than 15 carbon atoms) are immobilized in the sediment matrix. Large organic molecules such as DNA are likely to adhere to solid-phase sediments (particles, particulate organic matter). Industrially derived organic molecules (PAHs, PCBs) show marked patterns of increase in lake sediments at locally appropriate time-horizons, with no evidence of vertical leaching [61,62].

Despite the important work left to be done, our study clearly shows significant changes in the genetic composition of plant communities in Holocene lake sediments (Figure 6). Indeed, the significant increase in Fagaceae sequences in recent sediments matches the increased representation of Fagaceous macrofossils in those sediments [56].

## Authors' contributions

JSM, STJ, and HNP conceived of the study. LLA-C, JSM, HNP, STJ, MK and CL wrote the manuscript, conducted the molecular studies, and analyzed the data. STJ extracted and processed the macrofossils. HNP and MK developed the protocols and supervised all laboratory activities. All authors read and approved the final manuscript

## Supplementary Material

Additional file 1 **Sample information**. Sample type, DNA quantity, and haplotype ID for each sample analyzed in this study. Click here for file
