# Supplementary material for: Ancient DNA from lake sediments: Bridging the gap between paleoecology and genetics
Source: BMC Evol Biol. 2011 Jan 27;11:30. doi: 10.1186/1471-2148-11-30 (PMC3041685; doi:10.1186/1471-2148-11-30)
Supplement: Additional file 1 — Sample information. Sample type, DNA quantity, and haplotype ID for each sample analyzed in this study. [file 1471-2148-11-30-S1.DOC]

Additional File 1. DNA quantity (# copies 5 μl^-1^). Sequences were obtained either by direct sequencing or by sequencing col-PCR products. Macrofossil species (Bet = *Betula allegheniensis*, Fagr1, Fagr2 = *Fagus grandifolia*, Sap = Sapindaceae, Qr = *Quercus rubra*, and bulk = bulk sediments). Age is in calibrated years before present, lake locations correspond with Figure 1, Organ represents macrofossil type, or bulk sediments. Dominant sequence indicates whether 50% or more of unambiguous sequences came from the corresponding macrofossil, and sequence types fall into either 3 taxonomic groups *Betula*ceae, *Fagaceae (including a second Fagus grandifolia haplotype* and *Quercus rubra)*, or *Sapindaceae* (see methods). Other sequences were classified as U (unknown origin) or N (the presence of an ambiguous base). Haplotypes are listed in order if they were obtained directly/cloned.

| **Quant. (copies)** | **Direct or Cloned Seqs.** | **Species** | **Age** | **Lake** | **Organ** | **Dom Seq** | **Bet** | **Fagr 1** | **Fagr 2 2** | **Sap** | **1U** | **2U** | **3U** | **4U** | **5U** | **6U** | **7U** | **8U** | **9U** | **10U** | **11U** | **12U** | **N** |
| --- | --- | --- | --- | --- | --- | --- | --- | --- | --- | --- | --- | --- | --- | --- | --- | --- | --- | --- | --- | --- | --- | --- | --- |
| **1.28E+01** | Direct (2) Clone (2) | Bet | 502 | Ackerman | seed | Y | 2/2 |  |  |  |  |  |  |  |  |  |  |  |  |  |  |  |  |
| **N/A** | N/A (No Amp.) | Fagr | 502 | Ackerman | budscale |  |  |  |  |  |  |  |  |  |  |  |  |  |  |  |  |  |  |
| **N/A** | Direct (1) | Fagr | 502 | Ackerman | budscale | Y |  |  | 1/0 |  |  |  |  |  |  |  |  |  |  |  |  |  |  |
| **N/A** | N/A (No Amp.) | Fagr | 502 | Ackerman | budscale |  |  |  |  |  |  |  |  |  |  |  |  |  |  |  |  |  |  |
| **1.26** | Direct (2) Clone (7) | Fagr | 502 | Ackerman | budscale | Y |  |  | 2/7 |  |  |  |  |  |  |  |  |  |  |  |  |  |  |
| **N/A** | N/A (No Amp.) | Sap | 1108 | Ackerman | samara |  |  |  |  |  |  |  |  |  |  |  |  |  |  |  |  |  |  |
| **N/A** | Direct (1) Clone (2) | Sap | 1108 | Ackerman | samara | N |  | 0/2 |  |  | 1/0 |  |  |  |  |  |  |  |  |  |  |  |  |
| **3.34** | Direct (1) | Sap | 1108 | Ackerman | samara | N |  | 1/0 |  |  |  |  |  |  |  |  |  |  |  |  |  |  |  |
| **N/A** | N/A (No Amp.) | Bet | 1108 | Ackerman | seed |  |  |  |  |  |  |  |  |  |  |  |  |  |  |  |  |  |  |
| **7.61E-01** | Direct (1) Clone (1) | Bet | 1108 | Ackerman | seed | Y | 1/1 |  |  |  |  |  |  |  |  |  |  |  |  |  |  |  |  |
| **1.41** | Direct (2) Clone (6) | Bet | 1108 | Ackerman | bract | N | 1/2 |  |  |  |  | 0/3 |  |  |  |  |  |  |  |  |  |  | 1/1 |
| **5.47E+01** | Direct (3) Clone (5) | Bet | 1108 | Ackerman | leaf | Y | 1/3 |  |  |  |  |  | 0/1 |  |  |  |  |  |  |  |  |  | 2/1 |
| **2.63** | Direct (1), Clone (6) | Fagr | 1108 | Ackerman | budscale | Y |  |  | 1/6 |  |  |  |  |  |  |  |  |  |  |  |  |  |  |
| **N/A** | N/A (No Amp.) | Fagr | 1108 | Ackerman | budscale |  |  |  |  |  |  |  |  |  |  |  |  |  |  |  |  |  |  |
| **N/A** | N/A (No Amp.) | Fagr | 1108 | Ackerman | budscale |  |  |  |  |  |  |  |  |  |  |  |  |  |  |  |  |  |  |
| **4.02E-02** | Direct (2) Clone (1) | Fagr | 1108 | Ackerman | budscale | Y |  | 1/0 | 0/1 |  |  |  |  |  |  |  |  |  |  |  |  |  | 1/0 |
| **3.69** | Direct (2) Clone (13) | Fagr | 1108 | Ackerman | leaf | N | 1/12 |  |  |  |  |  |  |  |  |  |  |  |  |  |  |  | 1/1 |
| **1.54** | Direct (2) Clone (9) | Fagr | 1108 | Ackerman | budscale | N | 0/9 |  |  |  |  |  |  |  |  |  |  |  |  |  |  |  | 2/0 |
| **N/A** | Direct (1) | Bet | 1213 | Ackerman | seed | Y | 1/0 |  |  |  |  |  |  |  |  |  |  |  |  |  |  |  |  |
| **N/A** | Direct (1) Clone (2) | bulk | 1319 | Ackerman | \ |  |  | 0/2 | 1/0 |  |  |  |  |  |  |  |  |  |  |  |  |  |  |
| **9.28E+00** | Direct (2), Clone (8) | Bet | 1340 | Ackerman | seed | Y | 1/7 |  |  |  |  | 0/1 |  |  |  |  |  |  |  |  |  |  | 1/0 |
| **1.37E-01** | Direct (1) | Bet | 1340 | Ackerman | seed | N |  |  |  |  |  |  |  |  |  |  |  |  |  |  |  |  | 1/0 |
| **5.37E-01** | Direct (1) Clone (1) | Bet | 1340 | Ackerman | bract | Y | 0/1 |  |  |  |  |  |  |  |  |  |  |  |  |  |  |  | 1/0 |
| **2.35** | Direct (1) | Fagr | 1340 | Ackerman | budscale | Y |  | 1/0 |  |  |  |  |  |  |  |  |  |  |  |  |  |  |  |
| **N/A** | N/A (No Amp.) | Bet | 1425 | Ackerman | seed |  |  |  |  |  |  |  |  |  |  |  |  |  |  |  |  |  |  |
| **N/A** | N/A (No Amp.) | Bet | 1425 | Ackerman | seed |  |  |  |  |  |  |  |  |  |  |  |  |  |  |  |  |  |  |
| **1.19E-01** | Direct (2) Clone (4) | Fagr | 1425 | Ackerman | budscale | Y |  | 0/4 |  |  |  |  |  |  |  |  |  |  |  |  |  |  | 2/0 |
| **N/A** | Direct (1) | Bet | 1531 | Ackerman | seed | N |  |  |  |  |  |  |  |  |  |  |  |  |  |  |  |  | 1/0 |
| **N/A** | N/A (No Amp.) | Qr | 1531 | Ackerman | budscale |  |  |  |  |  |  |  |  |  |  |  |  |  |  |  |  |  |  |
| **4.14E-01** | Direct (1) Clone (1) | Bet | 1700 | Ackerman | seed | Y | 1/1 |  |  |  |  |  |  |  |  |  |  |  |  |  |  |  |  |
| **1.74** | Direct (1) Clone (2) | Bet | 1700 | Ackerman | seed | Y | 1/2 |  |  |  |  |  |  |  |  |  |  |  |  |  |  |  |  |
| **N/A** | N/A (No Amp.) | Bet | 1700 | Ackerman | bract |  |  |  |  |  |  |  |  |  |  |  |  |  |  |  |  |  |  |
|  |  |  |  |  |  |  |  |  |  |  |  |  |  |  |  |  |  |  |  |  |  |  |  |
|  |  |  |  |  |  |  |  |  |  |  |  |  |  |  |  |  |  |  |  |  |  |  |  |
| **Quant. (copies)** | **Direct or Cloned Seqs.** | **Species** | **Age** | **Lake** | **Organ** | **Dom Seq** | **Bet** | **Fagr 1** | **Fagr 2** | **Sap** | **1U** | **2U** | **3U** | **4U** | **5U** | **6U** | **7U** | **8U** | **9U** | **10U** | **11U** | **12U** | **N** |
| **N/A** | Direct (1) Clone (8) | Bet | 1742 | Ackerman | seed | Y | 0/8 |  |  |  |  |  |  |  |  |  |  |  |  |  |  |  | 1/0 |
| **N/A** | N/A (No Amp.) | Bet | 1742 | Ackerman | seed |  |  |  |  |  |  |  |  |  |  |  |  |  |  |  |  |  |  |
| **5.25E-01** | Direct (1) | Bet | 1848 | Ackerman | seed | Y | 1/0 |  |  |  |  |  |  |  |  |  |  |  |  |  |  |  |  |
| **3.50E-02** | Direct (3) | Bet | 1848 | Ackerman | seed | Y | 1/0 |  |  |  |  |  |  | 1/0 |  |  |  |  |  |  |  |  | 1/0 |
| **N/A** | N/A (No Amp.) | Bet | 1953 | Ackerman | seed |  |  |  |  |  |  |  |  |  |  |  |  |  |  |  |  |  |  |
| **N/A** | N/A (No Amp.) | Bet | 3440 | Ackerman | seed |  |  |  |  |  |  |  |  |  |  |  |  |  |  |  |  |  |  |
| **N/A** | N/A (No Amp.) | Bet | 3440 | Ackerman | seed |  |  |  |  |  |  |  |  |  |  |  |  |  |  |  |  |  |  |
| **N/A** | N/A (No Amp.) | Qr | 4740 | Ackerman | budscale |  |  |  |  |  |  |  |  |  |  |  |  |  |  |  |  |  |  |
| **N/A** | N/A (No Amp.) | Qr | 4740 | Ackerman | budscale |  |  |  |  |  |  |  |  |  |  |  |  |  |  |  |  |  |  |
| **2.03E+02** | Direct (2) Clone (4) | Bulk | 89 | Canyon | \ |  |  | 0/3 |  |  |  |  |  |  | 0/1 |  |  |  |  |  |  |  | 2/0 |
| **5.73E+01** | Direct (2) | Qr | 667 | Canyon | leaf | Y |  | 2/0 |  |  |  |  |  |  |  |  |  |  |  |  |  |  |  |
| **7.61E+04** | Direct (2) | Qr | 838 | Canyon | budscale | Y |  | 2/0 |  |  |  |  |  |  |  |  |  |  |  |  |  |  |  |
| **7.97E+02** | Direct (3) | Bet | 711 | Hells Kit. | seed | Y | 2/0 |  |  |  |  |  |  |  |  |  |  |  |  |  |  |  | 1/0 |
| **2.32E+01** | Direct (3) | Bet | 806 | Hells Kit. | seed | Y | 2/0 |  |  |  |  |  |  |  |  |  |  |  |  |  |  |  | 1/0 |
| **N/A** | N/A (No Amp.) | Bet | 993 | Hells Kit. | seed |  |  |  |  |  |  |  |  |  |  |  |  |  |  |  |  |  |  |
| **N/A** | N/A (No Amp.) | Bet | 2575 | South Kratt | seed |  |  |  |  |  |  |  |  |  |  |  |  |  |  |  |  |  |  |
| **N/A** | N/A (No Amp.) | Bet | 2921 | South Kratt | seed |  |  |  |  |  |  |  |  |  |  |  |  |  |  |  |  |  |  |
| **N/A** | N/A (No Amp.) | Qr | 6685 | South Kratt | budscale |  |  |  |  |  |  |  |  |  |  |  |  |  |  |  |  |  |  |
| **N/A** | N/A (No Amp.) | Qr | 6685 | South Kratt | budscale |  |  |  |  |  |  |  |  |  |  |  |  |  |  |  |  |  |  |
| **N/A** | N/A (No Amp.) | Qr | 6685 | South Kratt | budscale |  |  |  |  |  |  |  |  |  |  |  |  |  |  |  |  |  |  |
| **3.00E-01** | Direct (2) Clone (14) | Bet | 325 | Tower | seed | Y | 2/14 |  |  |  |  |  |  |  |  |  |  |  |  |  |  |  |  |
| **2.11** | Direct (2) Clone (12) | Fagr | 325 | Tower | budscale | Y |  | 2/12 |  |  |  |  |  |  |  |  |  |  |  |  |  |  |  |
| **3.36E+03** | Direct (3) | Fagr | 325 | Tower | budscale | Y |  | 3/0 |  |  |  |  |  |  |  |  |  |  |  |  |  |  |  |
| **N/A** | N/A (No Amp.) | Fagr | 325 | Tower | budscale |  |  |  |  |  |  |  |  |  |  |  |  |  |  |  |  |  |  |
| **N/A** | N/A (No Amp.) | Fagr | 507 | Tower | budscale |  |  |  |  |  |  |  |  |  |  |  |  |  |  |  |  |  |  |
| **N/A** | Clone (4) | Bet | 766 | Tower | seed | N |  | 0/4 |  |  |  |  |  |  |  |  |  |  |  |  |  |  |  |
| **3.35E+02** | Direct (3) Clone (7) | Fagr | 766 | Tower | budscale | Y |  | 3/7 |  |  |  |  |  |  |  |  |  |  |  |  |  |  |  |
| **1.39E+02** | Direct (3) | Bet | 896 | Tower | seed | Y | 3/0 |  |  |  |  |  |  |  |  |  |  |  |  |  |  |  |  |
| **2.02E-02** | Direct (2) | Sap | 1059 | Tower | budscale | N |  | 2/0 |  |  |  |  |  |  |  |  |  |  |  |  |  |  |  |
| **N/A** | N/A (No Amp.) | Sap | 1059 | Tower | budscale |  |  |  |  |  |  |  |  |  |  |  |  |  |  |  |  |  |  |
| **N/A** | N/A (No Amp.) | Fagr | 1059 | Tower | budscale |  |  |  |  |  |  |  |  |  |  |  |  |  |  |  |  |  |  |
| **3.89E+01** | Direct (3) | Bet | 1206 | Tower | catkin bract | Y | 3/0 |  |  |  |  |  |  |  |  |  |  |  |  |  |  |  |  |
| **9.35E-01** | Direct (2) | Fagr | 1206 | Tower | budscale | Y |  | 2/0 |  |  |  |  |  |  |  |  |  |  |  |  |  |  |  |
| **N/A** | N/A (No Amp.) | Fagr | 1206 | Tower | budscale |  |  |  |  |  |  |  |  |  |  |  |  |  |  |  |  |  |  |
| **N/A** | N/A (No Amp.) | Fagr | 1206 | Tower | budscale |  |  |  |  |  |  |  |  |  |  |  |  |  |  |  |  |  |  |
| **N/A** | N/A (No Amp.) | Fagr | 1206 | Tower | budscale |  |  |  |  |  |  |  |  |  |  |  |  |  |  |  |  |  |  |
| **N/A** | N/A (No Amp.) | Fagr | 1206 | Tower | budscale |  |  |  |  |  |  |  |  |  |  |  |  |  |  |  |  |  |  |
| **N/A** | N/A (No Amp.) | Fagr | 1206 | Tower | budscale |  |  |  |  |  |  |  |  |  |  |  |  |  |  |  |  |  |  |
|  |  |  |  |  |  |  |  |  |  |  |  |  |  |  |  |  |  |  |  |  |  |  |  |
| **Quant. (copies)** | **Direct or Cloned Seqs.** | **Species** | **Age** | **Lake** | **Organ** | **Dom Seq** | **Bet** | **Fagr 1** | **Fagr 2** | **Sap** | **1U** | **2U** | **3U** | **4U** | **5U** | **6U** | **7U** | **8U** | **9U** | **10U** | **11U** | **12U** | **N** |
| **6.25E-02** | Direct (2) Clone (1) | Bet | 1353 | Tower | seed | Y | 0/1 |  |  |  |  |  |  |  |  | 1/0 |  |  |  |  |  |  | 1/0 |
| **1.83E+01** | Direct (3) Clone (7) | Bet | 1353 | Tower | seed | Y | 3/3 |  |  |  |  |  |  |  |  |  | 0/3 |  |  |  |  |  | 0/1 |
| **2.71** | Direct (1) Clone (7) | Bet | 1353 | Tower | seed | Y | 0/4 |  |  |  |  |  |  |  |  |  |  | 0/3 |  |  |  |  | 1/0 |
| **N/A** | N/A (No Amp.) | Bet | 1353 | Tower | seed |  |  |  |  |  |  |  |  |  |  |  |  |  |  |  |  |  |  |
| **2.49** | Direct (1) Clone (2) | Fagr | 1353 | Tower | budscale | Y |  | 1/2 |  |  |  |  |  |  |  |  |  |  |  |  |  |  |  |
| **N/A** | N/A (No Amp.) | Fagr | 1353 | Tower | budscale |  |  |  |  |  |  |  |  |  |  |  |  |  |  |  |  |  |  |
| **2.88** | Direct (4) | Bet | 1500 | Tower | seed | Y | 2/0 |  |  |  |  |  |  |  |  |  |  |  |  |  |  |  | 2/0 |
| **3.69** | Direct (3) Clone (2) | Bet | 1500 | Tower | seed | Y | 1/2 |  |  |  |  |  |  |  |  |  |  |  | 1/0 | 1/0 |  |  |  |
| **1.02E+01** | Direct (2) Clone (13) | Bet | 1646 | Tower | seed | Y | 2/11 |  |  |  |  | 0/1 |  |  |  |  |  |  |  |  | 0/1 |  |  |
| **2.35E+01** | Direct (4) | Bet | 1646 | Tower | seed | Y | 4/0 |  |  |  |  |  |  |  |  |  |  |  |  |  |  |  |  |
| **1.37E+02** | Direct (4) | Bet | 1646 | Tower | seed | Y | 4/0 |  |  |  |  |  |  |  |  |  |  |  |  |  |  |  |  |
| **3.82E+01** | Direct (3) | Bet | 1646 | Tower | seed | Y | 3/0 |  |  |  |  |  |  |  |  |  |  |  |  |  |  |  |  |
| **5.15E+02** | Direct (8) Clone (4) | Qr | 1646 | Tower | budscale | Y |  | 8/4 |  |  |  |  |  |  |  |  |  |  |  |  |  |  |  |
| **1.21E-01** | Direct (5) Clone (13) | Bulk | 1720 | Tower | \ |  | 0/5 |  |  |  |  |  |  |  |  |  |  |  |  |  |  | 0/8 | 5/0 |
| **4.10E-01** | N/A (No Amp.) | Bulk | 1865 | Tower | \ |  |  |  |  |  |  |  |  |  |  |  |  |  |  |  |  |  |  |
| **2.89** | Direct (1) Clone (9) | Bet | 1971 | Tower | seed | Y | 0/7 |  |  |  |  |  |  |  |  |  |  |  |  |  | 0/2 |  | 1/0 |
| **N/A** | N/A (No Amp.) | Bet | 1971 | Tower | seed |  |  |  |  |  |  |  |  |  |  |  |  |  |  |  |  |  |  |
| **2.4** | N/A (No Amp.) | Bet | 1971 | Tower | seed |  |  |  |  |  |  |  |  |  |  |  |  |  |  |  |  |  |  |
| **6.33** | Direct (4) Clone (8) | Bet | 1971 | Tower | seed | Y | 2/7 |  |  |  |  | 0/1 |  |  |  |  |  |  |  |  |  |  | 2/0 |
| **N/A** | N/A (No Amp.) | Bet | 1971 | Tower | seed |  |  |  |  |  |  |  |  |  |  |  |  |  |  |  |  |  |  |
| **N/A** | N/A (No Amp.) | Bet | 1971 | Tower | seed |  |  |  |  |  |  |  |  |  |  |  |  |  |  |  |  |  |  |
| **9.23E-01** | Direct (2) Clone (2) | Bet | 1971 | Tower | seed | Y | 1/2 |  |  |  |  |  |  |  |  |  |  |  |  |  |  |  | 1/0 |
| **9.19** | Direct (3) Clone (7) | Bet | 1971 | Tower | seed | Y | 2/7 |  |  |  |  |  |  |  |  |  |  |  |  |  |  |  | 1/0 |
| **7.28E-01** | Direct (3) Clone (1) | Bet | 1971 | Tower | seed | Y | 1/1 |  |  |  |  |  |  |  |  |  |  |  |  |  |  |  | 2/0 |
| **1.96E-01** | Direct (1) Clone (8) | Bulk | 2040 | Tower | \ |  |  | 0/8 |  |  |  |  |  |  |  |  |  |  |  |  |  |  | 1/0 |
| **1.18** | Direct (3) | Bet | 2110 | Tower | seed | Y | 2/0 |  |  |  |  |  |  |  |  |  |  |  |  |  |  |  | 1/0 |
| **1.33** | Direct (2) | Bet | 2110 | Tower | seed | N |  |  |  |  |  |  |  |  |  |  |  |  |  |  |  |  | 2/0 |
| **N/A** | N/A (No Amp.) | Bet | 2540 | Tower | seed |  |  |  |  |  |  |  |  |  |  |  |  |  |  |  |  |  |  |
| **N/A** | N/A (No Amp.) | Bet | 2540 | Tower | seed |  |  |  |  |  |  |  |  |  |  |  |  |  |  |  |  |  |  |
| **N/A** | N/A (No Amp.) | Bet | 3050 | Tower | seed |  |  |  |  |  |  |  |  |  |  |  |  |  |  |  |  |  |  |
| **N/A** | N/A (No Amp.) | Bet | 3050 | Tower | seed |  |  |  |  |  |  |  |  |  |  |  |  |  |  |  |  |  |  |
| **N/A** | N/A (No Amp.) | Bet | 3180 | Tower | seed |  |  |  |  |  |  |  |  |  |  |  |  |  |  |  |  |  |  |
| **N/A** | N/A (No Amp.) | Qr | 3250 | Tower | budscale |  |  |  |  |  |  |  |  |  |  |  |  |  |  |  |  |  |  |
| **N/A** | N/A (No Amp.) | Bet | 3520 | Tower | bract |  |  |  |  |  |  |  |  |  |  |  |  |  |  |  |  |  |  |
| **N/A** | N/A (No Amp.) | Bet | 3520 | Tower | seed |  |  |  |  |  |  |  |  |  |  |  |  |  |  |  |  |  |  |
| **N/A** | N/A (No Amp.) | Bet | 3520 | Tower | bark |  |  |  |  |  |  |  |  |  |  |  |  |  |  |  |  |  |  |
| **N/A** | N/A (No Amp.) | Bet | 3520 | Tower | seed |  |  |  |  |  |  |  |  |  |  |  |  |  |  |  |  |  |  |
| **N/A** | N/A (No Amp.) | Bet | 3890 | Tower | seed |  |  |  |  |  |  |  |  |  |  |  |  |  |  |  |  |  |  |
|  |  |  |  |  |  |  |  |  |  |  |  |  |  |  |  |  |  |  |  |  |  |  |  |
| **Quant. (copies)** | **Direct or Cloned Seqs.** | **Species** | **Age** | **Lake** | **Organ** | **Dom Seq** | **Bet** | **Fagr 1** | **Fagr 2 2** | **Sap** | **1U** | **2U** | **3U** | **4U** | **5U** | **6U** | **7U** | **8U** | **9U** | **10U** | **11U** | **12U** | **N** |
| **N/A** | N/A (No Amp.) | Qr | 4450 | Tower | budscale |  |  |  |  |  |  |  |  |  |  |  |  |  |  |  |  |  |  |
| **2.17E-01** | Direct (1) | Qr | 4612 | Tower | budscale | N |  |  |  | 1/0 |  |  |  |  |  |  |  |  |  |  |  |  |  |
| **N/A** | N/A (No Amp.) | Qr | 4950 | Tower | budscale |  |  |  |  |  |  |  |  |  |  |  |  |  |  |  |  |  |  |
| **N/A** | N/A (No Amp.) | Qr | 5490 | Tower | budscale |  |  |  |  |  |  |  |  |  |  |  |  |  |  |  |  |  |  |
| **N/A** | N/A (No Amp.) | Qr | 5960 | Tower | budscale |  |  |  |  |  |  |  |  |  |  |  |  |  |  |  |  |  |  |
| **N/A** | N/A (No Amp.) | Qr | 6350 | Tower | budscale |  |  |  |  |  |  |  |  |  |  |  |  |  |  |  |  |  |  |
| **N/A** | Direct (1) | Qr | 6550 | Tower | budscale | N |  |  |  |  |  |  |  |  |  |  |  |  |  |  |  |  | 1/0 |
| **N/A** | N/A (No Amp.) | Qr | 6955 | Tower | budscale |  |  |  |  |  |  |  |  |  |  |  |  |  |  |  |  |  |  |
| **N/A** | N/A (No Amp.) | Sap | 2200 | Tower | seed |  |  |  |  |  |  |  |  |  |  |  |  |  |  |  |  |  |  |
| **3.90E-01** | Direct (1) Clone (9) | Bulk | 1864 | Trout | \ |  |  | 0/9 |  |  |  |  |  |  |  |  |  |  |  |  |  |  | 1/0 |
| **2.04** | Direct (2) Clone (4) | Bulk | 1913 | Trout | \ |  |  |  |  |  |  |  |  |  |  |  |  |  |  |  |  | 0/4 | 2/0 |
| **8.51E-01** | Direct (1) Clone (8) | Bulk | 3443 | Trout | \ |  | 0/8 |  |  |  |  |  |  |  |  |  |  |  |  |  |  |  | 1/0 |
| **N/A** | N/A (No Amp.) | Bet | 338 | Young | seed |  |  |  |  |  |  |  |  |  |  |  |  |  |  |  |  |  |  |
| **N/A** | N/A (No Amp.) | Fagr | 660 | Young | budscale |  |  |  |  |  |  |  |  |  |  |  |  |  |  |  |  |  |  |
| **N/A** | N/A (No Amp.) | Bet | 1133 | Young | seed |  |  |  |  |  |  |  |  |  |  |  |  |  |  |  |  |  |  |
| **N/A** | N/A (No Amp.) | Bet | 1451 | Young | seed |  |  |  |  |  |  |  |  |  |  |  |  |  |  |  |  |  |  |
| **N/A** | N/A (No Amp.) | Bet | 1793 | Young | seed |  |  |  |  |  |  |  |  |  |  |  |  |  |  |  |  |  |  |
| **N/A** | N/A (No Amp.) | Bet | 2945 | Young | seed |  |  |  |  |  |  |  |  |  |  |  |  |  |  |  |  |  |  |
